# High-Precision Identification of Sensory and Motor Branches of the Recurrent Laryngeal Nerve Via Autofluorescence System in Thyroid Surgery

**DOI:** 10.7759/cureus.80262

**Published:** 2025-03-08

**Authors:** Fernando Dip, Rene Aleman, Federico Marinelli, Javier Guiselli, Raul Rosenthal, Alberto Rancati, Diego Sinagra

**Affiliations:** 1 Department of General Surgery, University of Buenos Aires, Buenos Aires, ARG; 2 Department of General Surgery, Cleveland Clinic Florida, Weston, USA

**Keywords:** autofluorescence, navigational surgery, optical imaging, surgical enhancement, thyroidectomy

## Abstract

Recurrent laryngeal nerve (RLN) injury is a critical complication in thyroidectomy, with the severe sequelae of operation-related vocal cord palsies. The primary therapy following RLN injury includes voice therapy and surgical reintervention, both of which render subpar results paired with a long road to recovery. Despite the development of technical measures to prevent inadvertent operational injury of the RLN, its occurrence is still a concern. A newly developed handheld device with nerve autofluorescence technology has emerged as a visual aid tool for the intraoperative identification of nervous anatomical landmarks in thyroid surgery, showcasing promising initial findings. This study evaluates the efficacy of the aforementioned device in the intraoperative identification and differentiation of sensory and motor branches of the RLN. Sixteen patients undergoing thyroid surgery were included in this study, of which 16 RLNs and its branches were examined. Basic demographics, indication for thyroid surgery, and postoperative outcomes were identified. Multiple intraoperative images were analyzed through image processing software programs for the total number of nerves and branches, type of branch (e.g., sensory versus motor branches), near-ultraviolet (NUV) light intensity emitted by the nerve structures, and length and angular aperture of branches. The ability to prevent operation-related RLN injury was clinically evaluated at postoperative follow-up. Following analyses, no significant difference was observed between NUV light intensity (p=0.70) or structural length (p=0.18) between sensory and motor nerve branches of the RLN. This was further confirmed by fast Fourier transform (FFT) analyses and three-dimensional surface plots. No partial or total vocal cord palsies were recorded in the perioperative period, thus confirming the accuracy for intraoperative identification of the RLN and the preservation of structural integrity irrespective of surgical technique or type of branch (sensory or motor). Altogether, these findings highlight the potential of autofluorescence technology to enhance surgical precision, improve nerve preservation, and reduce the risk of nerve injury via safe surgical navigation in comparison to current intraoperative neuromonitoring systems limited to motor branch detection.

## Introduction

When performing thyroid surgery, the RLN is particularly vulnerable to inadvertent injury due to its relationship with neighboring anatomical landmarks [1]. Injuring the RLN can lead to unilateral or bilateral vocal cord palsy. Determining the true incidence of RLN injury can be challenging due to its under-diagnosis and strenuous surgical implications. Symptoms can be absent or minimal regardless of impaired vocal cord function. Around 30% to 50% of patients with vocal cord paralysis may be asymptomatic [2]. Nevertheless, the incidence of RLN injury and neighboring branches following thyroid surgery ranges from 14% to 58% [3,4]. The inception of intraoperative neuromonitoring (IONM) systems has facilitated RLN localization and relatively reduced postoperative injury rates. Albeit, this approach is inherently limited to the detection of motor branches, thereby neglecting sensory branches, which are critical for maintaining the structural and functional integrity of the nerve. In addition to its limitations, IONM is costly and user-dependent, increases operative time, is not entirely innocuous, has inferior specificity and sensitivity rates for nerve detection, and lacks visual feedback for intraoperative identification. While injury to the sensory branches can be less apparent, it withholds the potential to result in significant postoperative complications, including hemorrhage, hypoparathyroidism, RLN and superior laryngeal nerve injuries, Horner syndrome, and chyle leak [4,5]. Consequently, a novel handheld device has surfaced employing nerve autofluorescence technology to shift the paradigm of real-time nerve isolating identification of sensorial and motor nerve branches.

The inception of nerve autofluorescence resulted from the understanding of natural tissue spectroscopy and its near-ultraviolet (NUV) light emission [6]. The conceptualization of the Dendrite® (Gera, Germany) device gave way to its first clinical applications, where it displayed its capacity as an innocuous device, with navigational ease, safety, feasibility, and unmatched accuracy for precise nerve identification [7]. Implementing this novel device in routine procedures has provided a surpassing and comprehensive assessment of the RLN, all whilst addressing the limitations of traditional IONM systems. The real-time imagery feedback of this device offers enhancement of surgical precision and reduces morbidity of inadvertent nerve injury. Notwithstanding its potential, the ability of this technique to discern the type of nerve structures is yet to be determined. To this effect, the authors herein present, to the best knowledge, the first study analyzing the ability of the Dendrite® device to accurately identify and preserve sensory and motor branches of the RLN during thyroid surgery.

## Materials and methods

Following Institutional Review Board (IRB) approval, a retrospective observational study was conducted at a tertiary and highly specialized care center for endocrine surgery. Patients between 18 and 75 years of age undergoing thyroidectomy were included. The inclusion criteria included patients who presented a root base pathology posing a high risk for RLN injury including thyroid reoperation cases, significantly large goiter, and invasive thyroid neoplasms. Exclusion criteria included age outside the allocated range, pre-existing vocal cord palsy, or a history of radiation therapy. Prior to undergoing the procedure and study enrollment, verbal and written informed consent was obtained from the patients.

Technique

**Figure 1:**
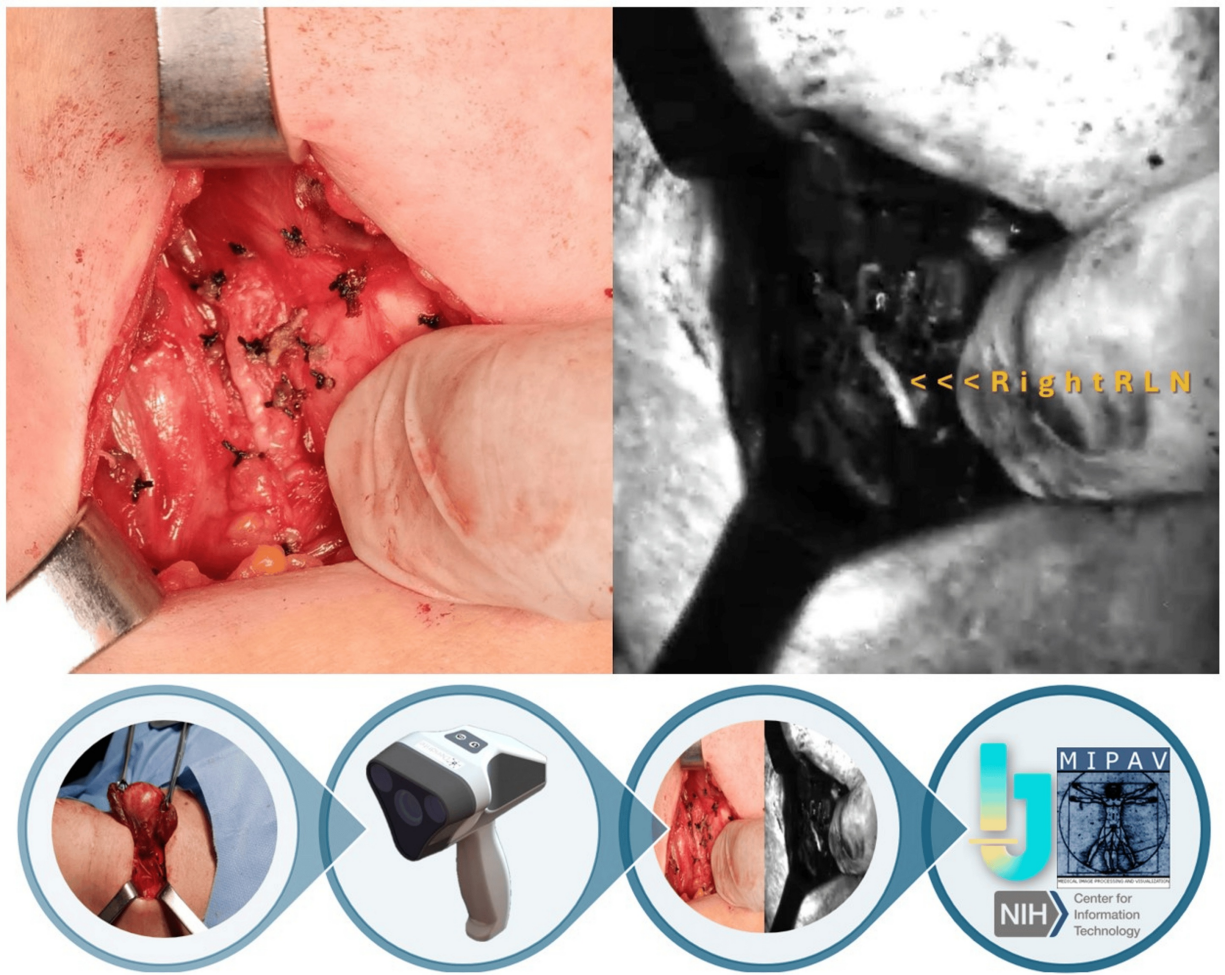
Approach Top left: conventional white light view during thyroidectomy. Top right: exteriorization of the thyroid gland visualized by the Dendrite® device and identification of the recurrent laryngeal nerve. Bottom: stepwise approach methodology. From left to right: incision and exposure of the thyroid gland followed by Dendrite® device identification of RLN with concomitant IONM of motor branches and subsequent image processing using ImageJ and MIPAV software programs. RLN, recurrent laryngeal nerve

Performance of thyroidectomies did not deviate from the standard technique. All procedures were performed in a two-step approach: following incision and before resection of the gland. Firstly, the Dendrite® device was used for intraoperative identification of all relevant and neighboring nerve structures. The device uses NUV light to excite neural structures, which emit autofluorescence captured and filtered by the device. At this time, cautious dissection of the gland was done under direct visualization of all nerve structures via nerve autofluorescence. Concomitantly, structural length, angular aperture, and NUV light intensity were measured and systematically recorded for each relevant structure. To differentiate sensory branches from motor branches, a nerve integrity monitoring (NIM™ 3.0, Medtronic, Minneapolis, MN) system was integrated into the procedure to confirm the presence of a motor branch and recorded for statistical comparison (Figure 1).

Data collection and statistical analysis

The data were prospectively collected as a retrospective chart review study in accordance with the inclusion criteria. At the time of gland dissection, multiple intraoperative images were taken and recorded for subsequent image processing. The open-source MIPAV and ImageJ software programs from the National Institutes of Health (NIH) Division of Computational Bioscience were used to create brightness-contrast histograms for NUV light intensity emitted by the nerve structures using pixels (px) as unit of measure for light intensity. Upon recording of NUV light intensity, multiple fast Fourier transform (FFT) analyses were performed to determine and confirm distribution of NUV light intensity subsequently represented into three-dimensional (3D) surface plots. Length of structures and angular aperture of each branch were recorded at the same time. After NIM system isolation of motor branches, data were allocated into two groups: group A (sensory branches) and group B (motor branches).

Data were presented as ranges, means, and standard deviations for continuous variables and as counts and percentages for categorical variables. Categorical data were presented using 𝜒2 and Fisher’s exact tests. Comparative analysis between NUV light intensity of group A and group B was performed via a paired t-test with a two-tailed distribution and a 95% confidence interval. Additionally, a correlation analysis was performed to determine the degree and strength of correlation between NUV light intensity and length among group A and group B. Statistical software R, version 4.4.2 for Windows (R Foundation for Statistical Computing, Vienna, Austria) was used for all statistical analyses, with an alpha level of 0.05.

## Results

A total of 16 patients were included in the study. The cohort was predominantly female, accounting for 88% (n=14) of the patients. Overall, the mean age was 49.7±13.9 years. The most common indication for thyroid surgery was thyroid cancer in 69% (n=11) of the cases, and the most performed procedure was total thyroidectomy in 75% (n=12) of all patients. The intraoperative identification of all relevant and neighboring nerve structures was achieved in 100% of the cases, rendering 100% sensitivity and 88% specificity rates for this cohort. Altogether, no device- or procedural-related complications were reported after an average follow-up time of 1,052.1±43.0 days. Table 1 summarizes the general demographics including general perioperative aspects.

**Table 1:** General demographics Avg, average; SD, standard deviation; RLN, recurrent laryngeal nerve; type 1 anatomical type, a single RLN branch entering the larynx; type 2 anatomical type; two RLN branches entering the larynx separately

N=16	N (%) or Avg±SD
Gender (female)	14 (88)
Age (years)	49.7±13.9
Indication for thyroidectomy	
Goiter	5 (31)
Thyroid cancer	11 (69)
Papillary thyroid cancer	11 (100)
OR time (mins)	67.9±8.6
Type of procedure	
Total thyroidectomy	12 (75)
Hemi-thyroidectomy	3 (19)
Parathyroidectomy	1 (6)
Successful nerve autofluorescence identification	16 (100)
Sensory branches	16 (100)
Motor branches	16 (100)
RLN anatomical type	
Type 1	6 (37)
Type 2	10 (63)
Accuracy	
Sensitivity	100%
Specificity	88%
Follow-up (days)	1,052.1±43.0
Complications	0 (0)

**Figure 2:**
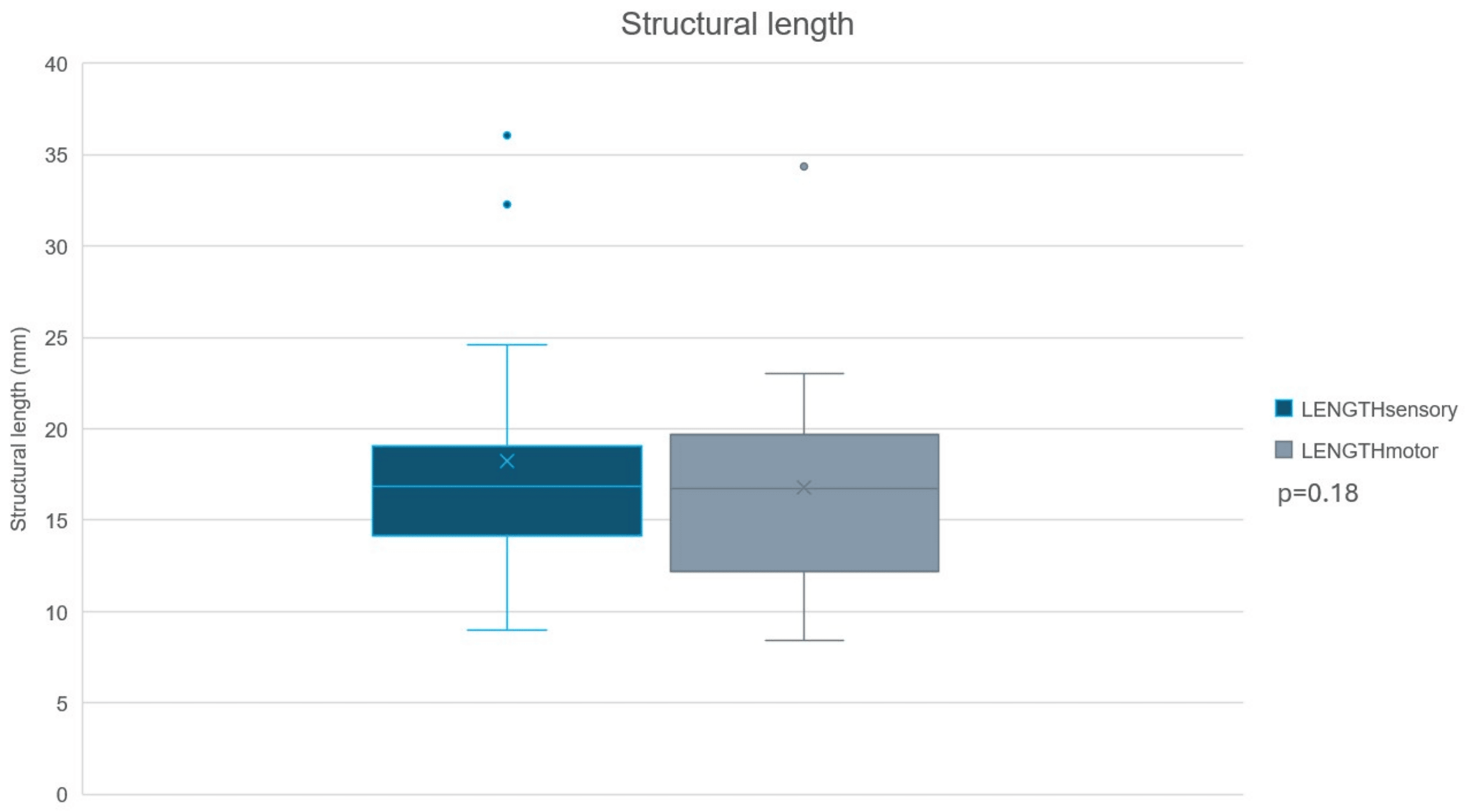
Structural length comparison Comparative figure of the mean structural length for group A (sensory branches) versus group B (motor branches).

**Figure 3:**
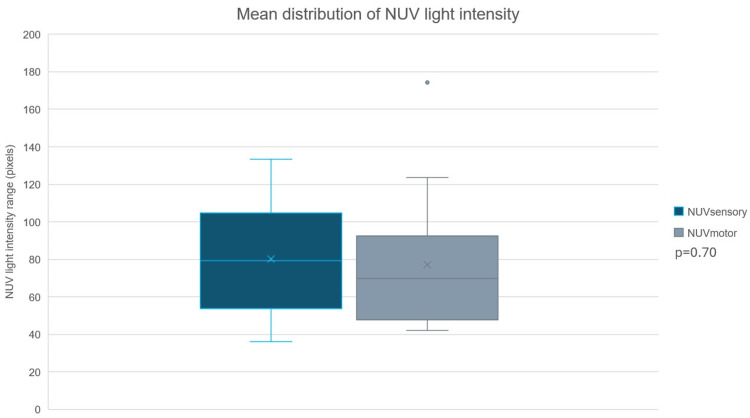
NUV light intensity comparison Comparative figure of the mean NUV light intensity for group A (sensory branches) versus group B (motor branches). NUV, near-ultraviolet

Following nerve identification, branches were isolated into sensory (group A) or motor (group B) branches with confirmatory IONM. Image processing software revealed a mean NUV light intensity of 80.2±29.2p x and 77.0±37.2 px for group A and group B, respectively. A paired t-test with a two-tailed distribution revealed no statistically significant difference in NUV light intensity between groups A and B (p=0.70; CI: 95%) (Figure 2). Similarly, the mean structural length of 18.3±7.1 mm and 16.8±6.4 mm for groups A and B, respectively, revealed no statistical significant difference (p=0.18; CI: 95%) (Figure 3). Additionally, correlation analyses revealed weak/positive and weak/negative degrees and strengths of correlation between NUV light intensity and structural length for group A and group B, respectively. These, however, were found to be not statistically significant (group A r value=0.14; p=0.26; group B r value=-0.044; p=0.72). Table 2 summarizes the general outcomes and collective image processing details.

**Table 2:** Outcomes (nerve image processing) P-value based on an alpha level of 0.05 and a confidence interval of 95%. AVG, average; RLN, recurrent laryngeal nerve; SD, standard deviation

N=16	Group A (Avg±SD)	Group B (Avg±SD)	p-Value
NUV light intensity (pixels)	80.2±29.2	77.0±37.2	0.70
Structural length (mm)	18.3±7.1	16.8±6.4	0.18
Aperture angle (degrees)	41.5±15.4	40.7±13.6	0.88
Correlation: light intensity versus length			
r value	0.14 (r≈0)	-0.044 (r≈0)	
Strength and direction	Weak and positive	Weak and negative	
Correlation p-value	0.26	0.72	
Postoperative outcome			
Postoperative RLN palsy	0 (0)	0 (0)	1

**Figure 4:**
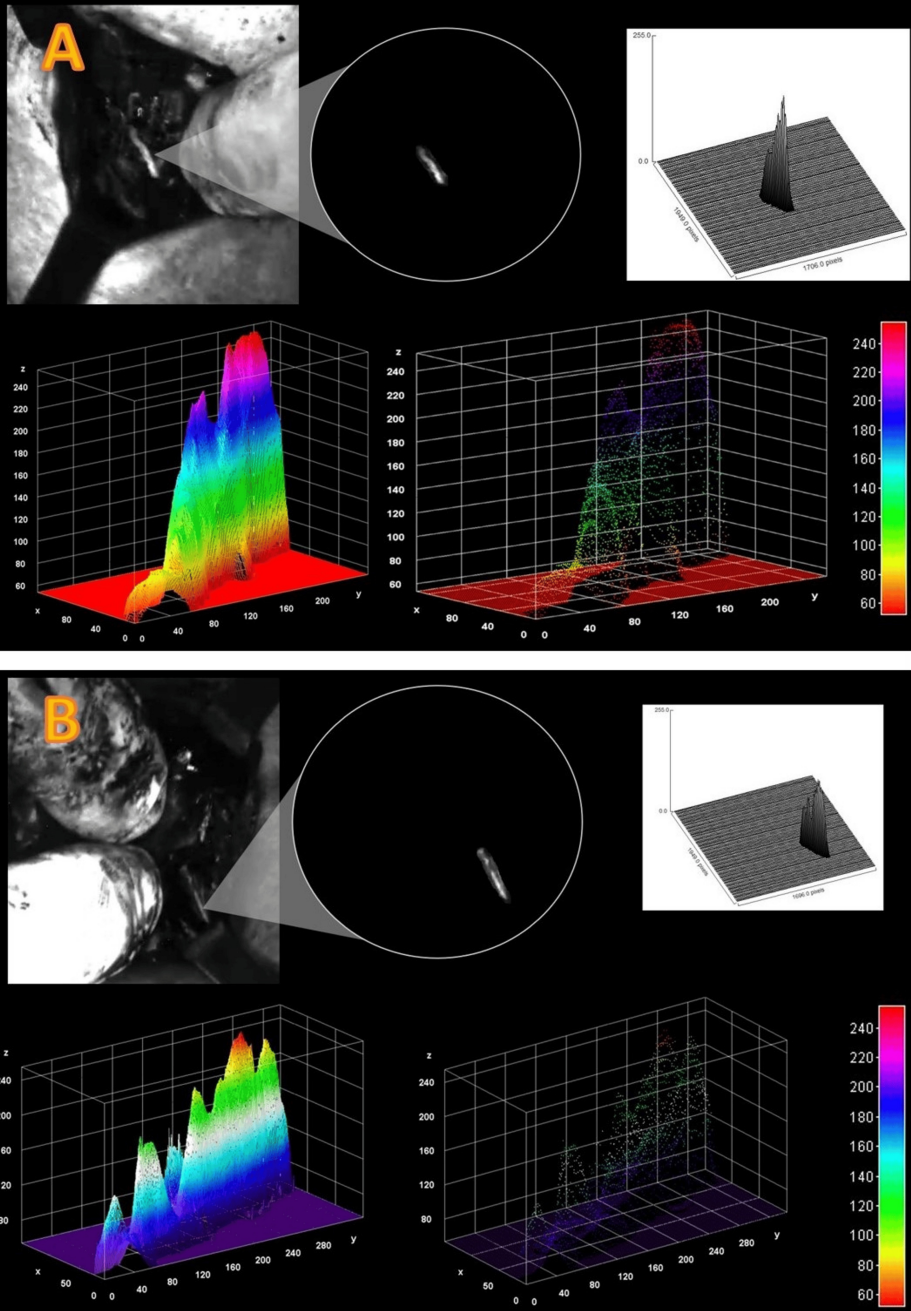
NUV light intensity distribution and 3D plots Top: 3D plot for the dimensional NUV light intensity distribution of a motor branch. Bottom: 3D plot for the dimensional NUV light intensity distribution of a sensory branch. NUV, near-ultraviolet

NUV light intensity distribution across all cases created brightness-contrast histograms to validate distribution through FFT analyses (high- and low-frequency sine waves that compile an image with high variability of light intensity) and showcased into 3D surface plots. The FFT analyses rendered an incidental finding for distance of light emitted. The 3D surface plots accounted for length, depth, and height of the neural structures. However, image processing isolated and collected data for emission of light. On average, a 10.0±2.8 mm nerve autofluorescence light intensity from both motor and sensitive branches was observed. Notwithstanding penetrance limitations of tissue artifact, this was set as the luminescence reach of RLN. All image processing analyses were performed in a retrospective fashion after data were collected (Figure 4).

## Discussion

This study demonstrates the effectiveness of autofluorescence imaging technology rendered by the Dendrite device. The technology enables an intraoperative visual aid tool for the identification and isolation of both sensory and motor branches of the RLN. This is further supported by the absence of vocal cord palsy after an average follow-up time of over two years, thus highlighting the potential of this technology to enhance surgical precision while ensuring nerve preservation. These results are particularly significant given the inclusion of high-risk cases, such as large goiters, reoperations, and invasive thyroid cancer.

As it stands, the comprehensive image processing rendered by the Dendrite® device validates the premise of its ability to accurately capture, identify, and isolate any and all nerve structures, including sensory and motor branches. The NUV light intensity between sensory and motor groups showed no significant difference, allowing for reliable surgical navigation that accounts for the preservation of all relevant and neighboring nerve structures. Said finding translates into the significant risk reduction rate of intraoperative nerve injuries during thyroid surgery. Moreover, when considering structural length, as well as degrees and strengths of correlation, the device has no visual limitations to convey excellent rates of specificity and sensibility for structural visualization. For contemporary surgical practice, the findings of this study suggest a significant shift in the dogma of thyroid surgery technique.

The inadvertent injury of the RLN is a dreaded complication of thyroid surgery with the potential to significantly impair quality of life. The occurrence of this complication is also the most common cause of surgical malpractice litigations [8]. Following thyroidectomy, the incidence of transient RLN injury ranges from 2% to 11%, whereas permanent injury occurs in 0.6% to 1.6% of cases [9-11]. Categorically, thyroidectomy for high-risk patients, such as those with malignant neoplasms, redo cases, and the presence of toxic or retrosternal goiter, is associated with a superimposed risk of nerve injury [12,13]. When nerve total vocal cord paralysis ensues, the aggregate morbidity requires the performance of emergent tracheotomy or acute surgical airway in around 50% of the compromised patients [14]. Consequently, IONM surfaced as a standard of care and routine technique as a contingency measure for RLN injury. Its application has been a continuous support to localize nerves and predict vocal cord function postoperatively. Moreover, the two-stage thyroidectomy performance concomitant with IONM prevents bilateral RLN total paralysis [15-17]. Nevertheless, this technique remains faulty.

A meta-analysis on the role of IONM revealed that its standard use during thyroid surgery resulted in RLN palsy rates of 4.9%. Of these, palsy was temporary in 64.6% and persistent in 35.4% of cases. In addition to these findings, the study deemed reoperation and total thyroidectomy as significant risk factors for the development of bilateral RLN palsy [18]. In general, IONM outcomes are beneficial yet imply an operational burden, including increasing operating room times, reliance on electrical stimulation to confirm motor nerve presence and function, limited indications, common reading errors, and complex algorithms for the loss of signal. Evidently, the clinical guidelines on IONM during thyroid and parathyroid surgery explicitly disclose these shortcomings [19]. Ultimately, these tools are not to be discouraged by operating surgeons as they convey sufficient benefits in the prevention of nerve injuries. Users, however, should be aware of its steep learning curve as it requires approximately 50 patients to achieve replicable results without abandoning altogether the possible complications of performing IONM [20]. On average, the use of IONM withholds a number of complications such as minor bleeding or bruising at the site of electrode placement, muscle tenderness, nerve trauma secondary to electrode placement, false-positive signals, and compartment syndrome. The factors that can contribute to these are inexperienced users, poor electrode placement, or surgical inexperience [21]. Given that IONM systems are limited to the isolation of motor branches by electrophysiological excitation, there are no tools to aid in the isolation or identification of sensory neural branches. The device implemented in the present study complements this underserved and inadvertent finding during thyroid surgery without compromising patient quality of life. Therefore, the presence of novel technology is encouraged as it has the potential to overcome the aforementioned drawbacks of cumbersome and relatively outdated technologies.

Firstly, as proven by this study, the Dendrite® device is entirely innocuous and requires no steep learning curve to replicate homogeneous safety, feasibility, and outcomes. Said premise is substantiated by the device’s high sensitivity and specificity rates despite high-risk and complex anatomical cases with the presence of patients with invasive neoplasms, significantly large goiter, and anatomical disruption in redo cases. In summary, structural properties, including length and NUV light intensity emitted by nerves, build on the ability of this technology to provide real-time identification, isolation, and, more importantly, preservation of these structures during performance of thyroid surgery. Secondly, the findings delivered by the Dendrite® device redirect the efforts of the surgical community to shift their attention to new technologies that broaden surgical capacities and improve surgical navigational skills.

Admittedly, this technique is still in its developing phase and warrants further research to aggregate the positive feedback on its benefits and potential applications [22,23]. Nonetheless, the authors recognize this study’s strengths and weaknesses. The initial findings drawn from this study fortify and expand validation of nerve autofluorescence technology. The rigorous understanding drawn from the applications of this technique strengthens its role as a surgical navigational and visual aid tool with the potential to positively alter the course of safe clinical practice. Due to its novelty, the authors also account for the inherent associated weaknesses. Although this study shows substantial and significant findings, it requires larger cohorts and comparative arms to strengthen the true role of this technique in RLN preservation surgery. In addition to this, the cost-effectiveness factor and mid- and long-term impact have yet to be determined and applied as clinical guidelines to develop surrounding nerve autofluorescence technologies, requiring larger cohort trials. In summary, the advancements in autofluorescence imaging could further improve surgical enhancement techniques and arguably result in virtually zero-risk factor procedures.

## Conclusions

The present study demonstrates the feasibility, safety, and efficacy of nerve autofluorescence technology as a visual aid and surgical navigational tool in thyroid surgery. Nerve autofluorescence technology through the Dendrite® device has proven to be an accurate and efficacious technique for the intraoperative identification of nerve structures irrespective of its sensory or motor nature, length, or angular aperture. Furthermore, the application of the aforementioned technology during standard technique procedures expeditiously provides imagery feedback for the operating surgeon without elevating the inadvertent risk for nerve injury seen in IONM tools. The absence of vocal cord palsy on postoperative evaluation strengthens the potential of this technology to improve surgical outcomes. Due to the relative novelty of this technology, larger studies are required to validate these initial findings. In addition, the exploration of this technology in other surgical subspecialties can refine its applications with the presupposition of becoming a standard-of-care tool to achieve unmatched surgical precision and ultimately preserve patient quality of life.
